# 4,4′-Di-*tert*-butyl-2,2′-[imidazolidine-1,3-diylbis(methyl­ene)]diphenol

**DOI:** 10.1107/S1600536813017157

**Published:** 2013-06-29

**Authors:** Augusto Rivera, Luz Stella Nerio, Michael Bolte

**Affiliations:** a Universidad Nacional de Colombia, Sede Bogotá, Facultad de Ciencias, Departamento de Química, Cra 30 No. 45-03, Bogotá, Código Postal 111321, Colombia; b Institut für Anorganische Chemie, J. W. Goethe-Universität Frankfurt, Max-von-Laue-Strasse 7, 60438 Frankfurt/Main, Germany

## Abstract

In the title compound, C_25_H_36_N_2_O_2_, the two *tert*-butyl-substituted benzene rings are inclined at an angle of 53.5 (3)° to one another. The imidazolidine ring has an envelope conformation with with one of the C atoms of the ethylene fragment as the flap. The structure displays two intra­molecular O—H⋯N hydrogen bonds that generate *S*(6) ring motifs. The crystal studied was a non-merohedral twin with a fractional contribution of 0.281(6) for the minor domain.

## Related literature

For related structures, see: Rivera *et al.* (2011 ▶, 2012*a* ▶,*b* ▶); Rivera, Nerio, Ríos-Motta, Fejfarová *et al.* (2012 ▶). For the use of the 2,2′-[imidazolidine-1,3-diylbis(methyl­ene)]diphenol system as a ligand in the synthesis of a variety of coordination compounds, see: Kober *et al.* (2012 ▶); Xu *et al.* (2007 ▶); Zhang *et al.* (2009 ▶). For the original synthesis of the title compound, see: Rivera *et al.* (1993 ▶). For hydrogen-bond motifs, see: Bernstein *et al.* (1995 ▶).
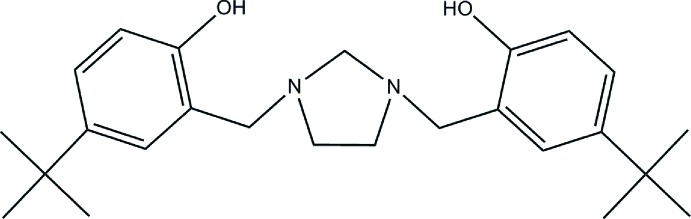


## Experimental

### 

#### Crystal data

C_25_H_36_N_2_O_2_
*M* *_r_* = 396.56
Monoclinic, 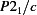

*a* = 21.0879 (16) Å
*b* = 6.2110 (4) Å
*c* = 17.9086 (16) Å
β = 109.168 (6)°
*V* = 2215.6 (3) Å^3^
*Z* = 4
Mo *K*α radiation
μ = 0.08 mm^−1^
*T* = 173 K
0.24 × 0.22 × 0.19 mm

#### Data collection

Stoe IPDS II two-circle diffractometer
22835 measured reflections
3909 independent reflections
3131 reflections with *I* > 2σ(*I*)
*R*_int_ = 0.101

#### Refinement

*R*[*F* ^2^ > 2σ(*F* ^2^)] = 0.119
*wR*(*F* ^2^) = 0.324
*S* = 1.13
3909 reflections
272 parameters
H atoms treated by a mixture of independent and constrained refinement
Δρ_max_ = 0.42 e Å^−3^
Δρ_min_ = −0.39 e Å^−3^

### 

Data collection: *X-AREA* (Stoe & Cie, 2001 ▶); cell refinement: *X-AREA*; data reduction: *XRED-32* (Stoe & Cie, 2001 ▶); program(s) used to solve structure: *SHELXS97* (Sheldrick, 2008 ▶); program(s) used to refine structure: *SHELXL97* (Sheldrick, 2008 ▶); molecular graphics: *DIAMOND* (Brandenburg, 2006 ▶); software used to prepare material for publication: *SHELXL97*.

## Supplementary Material

Crystal structure: contains datablock(s) I, global. DOI: 10.1107/S1600536813017157/sj5337sup1.cif

Structure factors: contains datablock(s) I. DOI: 10.1107/S1600536813017157/sj5337Isup2.hkl

Supplementary material file. DOI: 10.1107/S1600536813017157/sj5337Isup3.cml

Additional supplementary materials:  crystallographic information; 3D view; checkCIF report

## Figures and Tables

**Table 1:** Hydrogen-bond geometry (Å, °)

*D*—H⋯*A*	*D*—H	H⋯*A*	*D*⋯*A*	*D*—H⋯*A*
O1—H1⋯N1	1.00 (10)	1.70 (10)	2.655 (7)	157 (8)
O2—H2⋯N2	0.95 (8)	1.83 (8)	2.656 (7)	144 (7)

**Fig. 1.**
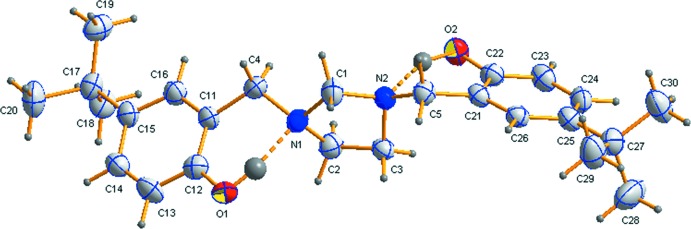
The molecular structure of the title compound, displacement ellipsoids are drawn at the 50% probability level. Intramolecular hydrogen bonds are drawn as dashed lines.

## supplementary crystallographic information

### Comment

2,2'-[imidazolidine-1,3-diylbis(methylene)]diphenol system has been used as ligand in the synthesis of a variety of coordination compounds, with potential applications in homogeneous catalysis (Kober *et al.*, 2012, Xu *et al*., 2007, Zhang *et al.*, 2009). The molecular structure and atom-numbering scheme for (I) are shown in Fig. 1. The imidazolidine ring adopts an envelope conformation, with C3 as the flap. The dihedral angle between aromatic rings is 53.5 (3)°. Its X-ray structure confirms the presence of two intramolecular hydrogen bonds between the phenolic hydroxyl groups and the imidazolidine nitrogen atoms with S(6) graph-set motifs (Bernstein *et al.*, 1995) (Table 1).

### Experimental

For the original synthesis of the title compound, see: Rivera *et al.* (1993). Single crystals suitable for X-ray diffraction were obtained from a mixture chloroform:methanol (1:1), by slow evaporation over 5 days at room temperature.

### Refinement

H atoms bonded to C were positioned geometrically, with C–H = 0.95–0.99 Å and constrained to ride on their parent atom, with *U*_iso_(H) = 1.5*U*_eq_(C) for methyl H atoms and 1.2*U*_eq_(C) for other H atoms. The crystal was a non-merohedral twin with a fractional contribution of 0.281 (6) for the minor domain. The twin law is (1 0 0.773/0 - 1 0/0 0 - 1). As a result of this twinning, the figures of merit are rather high. Nevertheless, the H atoms bonded to O could be located in a difference map and they could be freely refined.

### Crystal data

**Table:**

C_25_H_36_N_2_O_2_	*F*(000) = 864
*M**_r_* = 396.56	*D*_x_ = 1.189 Mg m^−^^3^
Monoclinic, *P*2_1_/*c*	Mo *K*α radiation, λ = 0.71073 Å
*a* = 21.0879 (16) Å	Cell parameters from 20905 reflections
*b* = 6.2110 (4) Å	θ = 2.1–26.0°
*c* = 17.9086 (16) Å	µ = 0.08 mm^−^^1^
β = 109.168 (6)°	*T* = 173 K
*V* = 2215.6 (3) Å^3^	Block, colourless
*Z* = 4	0.24 × 0.22 × 0.19 mm

### Data collection

**Table:**

Stoe IPDS II two-circle diffractometer	*R*_int_ = 0.101
Radiation source: Genix 3D IµS microfocus X-ray source	θ_max_ = 25.0°, θ_min_ = 2.1°
ω scans	*h* = −25→24
22835 measured reflections	*k* = −7→7
3909 independent reflections	*l* = −18→21
3131 reflections with *I* > 2σ(*I*)	

### Refinement

**Table:**

Refinement on *F*^2^	Hydrogen site location: mixed
Least-squares matrix: full	H atoms treated by a mixture of independent and constrained refinement
*R*[*F*^2^ > 2σ(*F*^2^)] = 0.119	*w* = 1/[σ^2^(*F*_o_^2^) + (0.0705*P*)^2^ + 10.9502*P*] where *P* = (*F*_o_^2^ + 2*F*_c_^2^)/3
*wR*(*F*^2^) = 0.324	(Δ/σ)_max_ < 0.001
*S* = 1.13	Δρ_max_ = 0.42 e Å^−^^3^
3909 reflections	Δρ_min_ = −0.39 e Å^−^^3^
272 parameters	Extinction correction: *SHELXL97* (Sheldrick, 2008), Fc^*^=kFc[1+0.001xFc^2^λ^3^/sin(2θ)]^-1/4^
0 restraints	Extinction coefficient: 0.037 (4)

### Special details

**Table:**

Geometry. All e.s.d.'s (except the e.s.d. in the dihedral angle between two l.s. planes) are estimated using the full covariance matrix. The cell e.s.d.'s are taken into account individually in the estimation of e.s.d.'s in distances, angles and torsion angles; correlations between e.s.d.'s in cell parameters are only used when they are defined by crystal symmetry. An approximate (isotropic) treatment of cell e.s.d.'s is used for estimating e.s.d.'s involving l.s. planes.
Refinement. Refined as a 2-component twin.

### Fractional atomic coordinates and isotropic or equivalent isotropic displacement parameters (Å^2^)

**Table:**

	*x*	*y*	*z*	*U*_iso_*/*U*_eq_	
O1	0.4712 (2)	0.0117 (8)	0.5876 (3)	0.0455 (12)	
H1	0.500 (5)	0.122 (16)	0.624 (6)	0.09 (3)*	
O2	0.6663 (2)	0.9590 (8)	0.7246 (3)	0.0455 (12)	
H2	0.635 (4)	0.845 (13)	0.716 (5)	0.06 (2)*	
N1	0.5256 (3)	0.3720 (9)	0.6606 (3)	0.0385 (13)	
N2	0.6261 (3)	0.5545 (8)	0.7268 (3)	0.0368 (13)	
C1	0.5734 (3)	0.4179 (13)	0.7397 (4)	0.0489 (18)	
H1A	0.5929	0.2827	0.7669	0.059*	
H1B	0.5508	0.4945	0.7724	0.059*	
C2	0.5537 (3)	0.4692 (12)	0.6021 (4)	0.0433 (16)	
H2A	0.5346	0.6139	0.5854	0.052*	
H2B	0.5453	0.3765	0.5549	0.052*	
C3	0.6280 (3)	0.4820 (11)	0.6489 (4)	0.0369 (14)	
H3A	0.6501	0.3398	0.6525	0.044*	
H3B	0.6513	0.5875	0.6256	0.044*	
C4	0.4565 (3)	0.4390 (11)	0.6528 (4)	0.0419 (16)	
H4A	0.4524	0.5964	0.6437	0.050*	
H4B	0.4483	0.4089	0.7031	0.050*	
C5	0.6896 (3)	0.5301 (11)	0.7933 (4)	0.0394 (15)	
H5A	0.7046	0.3785	0.7960	0.047*	
H5B	0.6813	0.5639	0.8434	0.047*	
C11	0.4026 (3)	0.3260 (10)	0.5857 (4)	0.0364 (14)	
C12	0.4124 (3)	0.1211 (11)	0.5592 (4)	0.0407 (15)	
C13	0.3586 (4)	0.0247 (11)	0.5019 (4)	0.0492 (18)	
H13	0.3648	−0.1113	0.4810	0.059*	
C14	0.2970 (4)	0.1210 (11)	0.4750 (4)	0.0449 (16)	
H14	0.2617	0.0508	0.4354	0.054*	
C15	0.2846 (3)	0.3197 (10)	0.5040 (4)	0.0399 (15)	
C16	0.3396 (3)	0.4209 (11)	0.5576 (4)	0.0421 (16)	
H16	0.3339	0.5608	0.5758	0.050*	
C17	0.2157 (3)	0.4268 (12)	0.4738 (4)	0.0420 (16)	
C18	0.2078 (4)	0.5451 (13)	0.3962 (4)	0.0518 (19)	
H18A	0.2141	0.4431	0.3574	0.078*	
H18B	0.2415	0.6596	0.4057	0.078*	
H18C	0.1628	0.6081	0.3758	0.078*	
C19	0.2058 (4)	0.5876 (14)	0.5326 (4)	0.055 (2)	
H19A	0.2104	0.5144	0.5825	0.082*	
H19B	0.1609	0.6510	0.5114	0.082*	
H19C	0.2396	0.7015	0.5417	0.082*	
C20	0.1589 (4)	0.2555 (15)	0.4574 (6)	0.066 (2)	
H20A	0.1629	0.1777	0.5063	0.099*	
H20B	0.1630	0.1537	0.4174	0.099*	
H20C	0.1152	0.3272	0.4381	0.099*	
C21	0.7443 (3)	0.6742 (10)	0.7855 (4)	0.0363 (14)	
C22	0.7299 (3)	0.8793 (10)	0.7516 (4)	0.0403 (15)	
C23	0.7822 (3)	1.0074 (11)	0.7463 (4)	0.0434 (16)	
H23	0.7731	1.1453	0.7222	0.052*	
C24	0.8475 (3)	0.9344 (11)	0.7760 (4)	0.0434 (16)	
H24	0.8825	1.0241	0.7715	0.052*	
C25	0.8639 (3)	0.7346 (11)	0.8122 (4)	0.0422 (16)	
C26	0.8101 (3)	0.6077 (10)	0.8153 (4)	0.0366 (14)	
H26	0.8194	0.4693	0.8390	0.044*	
C27	0.9369 (3)	0.6602 (12)	0.8458 (5)	0.0506 (18)	
C28	0.9627 (4)	0.613 (2)	0.7759 (6)	0.090 (4)	
H28A	0.9588	0.7433	0.7438	0.135*	
H28B	0.9359	0.4975	0.7432	0.135*	
H28C	1.0099	0.5687	0.7964	0.135*	
C29	0.9436 (4)	0.4555 (14)	0.8947 (6)	0.069 (2)	
H29A	0.9159	0.3417	0.8619	0.103*	
H29B	0.9286	0.4836	0.9401	0.103*	
H29C	0.9907	0.4094	0.9134	0.103*	
C30	0.9809 (4)	0.8335 (14)	0.8977 (6)	0.071 (3)	
H30A	0.9768	0.9669	0.8672	0.106*	
H30B	1.0278	0.7858	0.9156	0.106*	
H30C	0.9665	0.8595	0.9436	0.106*	

### Atomic displacement parameters (Å^2^)

**Table:**

	*U*^11^	*U*^22^	*U*^33^	*U*^12^	*U*^13^	*U*^23^
O1	0.044 (3)	0.040 (3)	0.055 (3)	0.004 (2)	0.020 (2)	−0.007 (2)
O2	0.039 (3)	0.036 (3)	0.055 (3)	0.004 (2)	0.007 (2)	0.006 (2)
N1	0.034 (3)	0.046 (3)	0.039 (3)	−0.001 (2)	0.017 (2)	0.001 (2)
N2	0.033 (3)	0.039 (3)	0.037 (3)	−0.003 (2)	0.011 (2)	−0.003 (2)
C1	0.039 (4)	0.055 (4)	0.053 (4)	−0.012 (3)	0.014 (3)	0.000 (4)
C2	0.039 (4)	0.047 (4)	0.041 (4)	0.003 (3)	0.010 (3)	0.001 (3)
C3	0.039 (3)	0.038 (3)	0.033 (3)	−0.004 (3)	0.011 (3)	−0.004 (3)
C4	0.036 (3)	0.042 (4)	0.051 (4)	−0.001 (3)	0.020 (3)	−0.008 (3)
C5	0.036 (3)	0.044 (4)	0.036 (3)	0.001 (3)	0.009 (3)	0.002 (3)
C11	0.037 (3)	0.032 (3)	0.041 (4)	−0.004 (3)	0.015 (3)	−0.003 (3)
C12	0.047 (4)	0.039 (4)	0.039 (3)	0.001 (3)	0.017 (3)	0.005 (3)
C13	0.060 (4)	0.032 (3)	0.052 (4)	−0.003 (3)	0.013 (4)	−0.005 (3)
C14	0.051 (4)	0.041 (4)	0.042 (4)	−0.008 (3)	0.014 (3)	−0.006 (3)
C15	0.040 (3)	0.038 (3)	0.042 (4)	−0.004 (3)	0.013 (3)	0.003 (3)
C16	0.038 (3)	0.036 (3)	0.050 (4)	−0.001 (3)	0.012 (3)	−0.001 (3)
C17	0.035 (3)	0.051 (4)	0.041 (4)	−0.001 (3)	0.014 (3)	0.006 (3)
C18	0.048 (4)	0.065 (5)	0.039 (4)	0.001 (4)	0.010 (3)	0.005 (3)
C19	0.048 (4)	0.068 (5)	0.046 (4)	0.013 (4)	0.012 (3)	0.000 (4)
C20	0.040 (4)	0.072 (5)	0.081 (6)	−0.012 (4)	0.011 (4)	0.006 (5)
C21	0.041 (3)	0.035 (3)	0.030 (3)	−0.004 (3)	0.009 (3)	−0.003 (3)
C22	0.042 (4)	0.035 (3)	0.044 (4)	0.000 (3)	0.013 (3)	−0.003 (3)
C23	0.055 (4)	0.036 (3)	0.037 (3)	−0.007 (3)	0.013 (3)	0.005 (3)
C24	0.042 (4)	0.047 (4)	0.043 (4)	−0.013 (3)	0.016 (3)	−0.003 (3)
C25	0.042 (4)	0.042 (4)	0.040 (4)	−0.005 (3)	0.010 (3)	−0.006 (3)
C26	0.036 (3)	0.036 (3)	0.035 (3)	0.001 (3)	0.008 (3)	−0.003 (3)
C27	0.040 (4)	0.055 (4)	0.052 (4)	−0.003 (3)	0.010 (3)	−0.007 (4)
C28	0.049 (5)	0.135 (10)	0.089 (7)	0.009 (6)	0.027 (5)	−0.027 (7)
C29	0.045 (4)	0.056 (5)	0.094 (7)	0.002 (4)	0.007 (4)	0.000 (5)
C30	0.055 (5)	0.055 (5)	0.085 (6)	−0.010 (4)	−0.001 (4)	−0.011 (5)

### Geometric parameters (Å, º)

**Table:**

O1—C12	1.357 (8)	C17—C18	1.533 (10)
O1—H1	1.00 (10)	C17—C20	1.556 (10)
O2—C22	1.360 (8)	C18—H18A	0.9800
O2—H2	0.95 (8)	C18—H18B	0.9800
N1—C1	1.473 (9)	C18—H18C	0.9800
N1—C4	1.478 (8)	C19—H19A	0.9800
N1—C2	1.490 (9)	C19—H19B	0.9800
N2—C1	1.476 (8)	C19—H19C	0.9800
N2—C5	1.479 (8)	C20—H20A	0.9800
N2—C3	1.479 (8)	C20—H20B	0.9800
C1—H1A	0.9900	C20—H20C	0.9800
C1—H1B	0.9900	C21—C26	1.377 (9)
C2—C3	1.517 (9)	C21—C22	1.401 (9)
C2—H2A	0.9900	C22—C23	1.389 (9)
C2—H2B	0.9900	C23—C24	1.378 (10)
C3—H3A	0.9900	C23—H23	0.9500
C3—H3B	0.9900	C24—C25	1.391 (10)
C4—C11	1.526 (9)	C24—H24	0.9500
C4—H4A	0.9900	C25—C26	1.397 (9)
C4—H4B	0.9900	C25—C27	1.529 (9)
C5—C21	1.503 (9)	C26—H26	0.9500
C5—H5A	0.9900	C27—C30	1.522 (10)
C5—H5B	0.9900	C27—C29	1.523 (12)
C11—C16	1.388 (9)	C27—C28	1.549 (12)
C11—C12	1.397 (9)	C28—H28A	0.9800
C12—C13	1.392 (10)	C28—H28B	0.9800
C13—C14	1.365 (10)	C28—H28C	0.9800
C13—H13	0.9500	C29—H29A	0.9800
C14—C15	1.397 (9)	C29—H29B	0.9800
C14—H14	0.9500	C29—H29C	0.9800
C15—C16	1.390 (9)	C30—H30A	0.9800
C15—C17	1.526 (9)	C30—H30B	0.9800
C16—H16	0.9500	C30—H30C	0.9800
C17—C19	1.514 (10)		
			
C12—O1—H1	101 (5)	C18—C17—C20	108.0 (6)
C22—O2—H2	110 (5)	C17—C18—H18A	109.5
C1—N1—C4	112.3 (5)	C17—C18—H18B	109.5
C1—N1—C2	107.0 (5)	H18A—C18—H18B	109.5
C4—N1—C2	115.3 (5)	C17—C18—H18C	109.5
C1—N2—C5	110.1 (5)	H18A—C18—H18C	109.5
C1—N2—C3	103.2 (5)	H18B—C18—H18C	109.5
C5—N2—C3	115.6 (5)	C17—C19—H19A	109.5
N1—C1—N2	105.8 (5)	C17—C19—H19B	109.5
N1—C1—H1A	110.6	H19A—C19—H19B	109.5
N2—C1—H1A	110.6	C17—C19—H19C	109.5
N1—C1—H1B	110.6	H19A—C19—H19C	109.5
N2—C1—H1B	110.6	H19B—C19—H19C	109.5
H1A—C1—H1B	108.7	C17—C20—H20A	109.5
N1—C2—C3	102.2 (5)	C17—C20—H20B	109.5
N1—C2—H2A	111.3	H20A—C20—H20B	109.5
C3—C2—H2A	111.3	C17—C20—H20C	109.5
N1—C2—H2B	111.3	H20A—C20—H20C	109.5
C3—C2—H2B	111.3	H20B—C20—H20C	109.5
H2A—C2—H2B	109.2	C26—C21—C22	119.3 (6)
N2—C3—C2	101.1 (5)	C26—C21—C5	119.5 (6)
N2—C3—H3A	111.5	C22—C21—C5	121.2 (6)
C2—C3—H3A	111.5	O2—C22—C23	118.7 (6)
N2—C3—H3B	111.5	O2—C22—C21	122.2 (6)
C2—C3—H3B	111.5	C23—C22—C21	119.1 (6)
H3A—C3—H3B	109.4	C24—C23—C22	120.0 (6)
N1—C4—C11	113.6 (5)	C24—C23—H23	120.0
N1—C4—H4A	108.8	C22—C23—H23	120.0
C11—C4—H4A	108.8	C23—C24—C25	122.5 (6)
N1—C4—H4B	108.8	C23—C24—H24	118.8
C11—C4—H4B	108.8	C25—C24—H24	118.8
H4A—C4—H4B	107.7	C24—C25—C26	116.2 (6)
N2—C5—C21	112.5 (5)	C24—C25—C27	121.2 (6)
N2—C5—H5A	109.1	C26—C25—C27	122.6 (6)
C21—C5—H5A	109.1	C21—C26—C25	122.9 (6)
N2—C5—H5B	109.1	C21—C26—H26	118.6
C21—C5—H5B	109.1	C25—C26—H26	118.6
H5A—C5—H5B	107.8	C30—C27—C29	108.5 (7)
C16—C11—C12	119.6 (6)	C30—C27—C25	111.1 (6)
C16—C11—C4	117.8 (6)	C29—C27—C25	111.7 (6)
C12—C11—C4	122.1 (6)	C30—C27—C28	108.7 (7)
O1—C12—C13	119.0 (6)	C29—C27—C28	108.4 (8)
O1—C12—C11	123.2 (6)	C25—C27—C28	108.4 (6)
C13—C12—C11	117.8 (6)	C27—C28—H28A	109.5
C14—C13—C12	121.5 (7)	C27—C28—H28B	109.5
C14—C13—H13	119.2	H28A—C28—H28B	109.5
C12—C13—H13	119.2	C27—C28—H28C	109.5
C13—C14—C15	121.9 (7)	H28A—C28—H28C	109.5
C13—C14—H14	119.0	H28B—C28—H28C	109.5
C15—C14—H14	119.0	C27—C29—H29A	109.5
C16—C15—C14	116.1 (6)	C27—C29—H29B	109.5
C16—C15—C17	121.9 (6)	H29A—C29—H29B	109.5
C14—C15—C17	121.8 (6)	C27—C29—H29C	109.5
C11—C16—C15	122.8 (6)	H29A—C29—H29C	109.5
C11—C16—H16	118.6	H29B—C29—H29C	109.5
C15—C16—H16	118.6	C27—C30—H30A	109.5
C19—C17—C15	112.0 (6)	C27—C30—H30B	109.5
C19—C17—C18	108.4 (6)	H30A—C30—H30B	109.5
C15—C17—C18	109.7 (6)	C27—C30—H30C	109.5
C19—C17—C20	108.0 (6)	H30A—C30—H30C	109.5
C15—C17—C20	110.6 (6)	H30B—C30—H30C	109.5
			
C4—N1—C1—N2	−123.4 (6)	C16—C15—C17—C19	−26.9 (9)
C2—N1—C1—N2	4.0 (7)	C14—C15—C17—C19	157.8 (7)
C5—N2—C1—N1	−154.4 (5)	C16—C15—C17—C18	93.5 (8)
C3—N2—C1—N1	−30.5 (7)	C14—C15—C17—C18	−81.8 (8)
C1—N1—C2—C3	23.1 (7)	C16—C15—C17—C20	−147.5 (7)
C4—N1—C2—C3	148.8 (5)	C14—C15—C17—C20	37.2 (9)
C1—N2—C3—C2	44.4 (6)	N2—C5—C21—C26	−148.4 (6)
C5—N2—C3—C2	164.6 (5)	N2—C5—C21—C22	34.6 (8)
N1—C2—C3—N2	−41.3 (6)	C26—C21—C22—O2	−176.7 (6)
C1—N1—C4—C11	−159.1 (6)	C5—C21—C22—O2	0.3 (10)
C2—N1—C4—C11	77.9 (7)	C26—C21—C22—C23	2.4 (9)
C1—N2—C5—C21	−177.0 (6)	C5—C21—C22—C23	179.4 (6)
C3—N2—C5—C21	66.5 (7)	O2—C22—C23—C24	177.5 (6)
N1—C4—C11—C16	−160.5 (6)	C21—C22—C23—C24	−1.6 (10)
N1—C4—C11—C12	27.4 (9)	C22—C23—C24—C25	−0.4 (11)
C16—C11—C12—O1	−175.5 (6)	C23—C24—C25—C26	1.6 (10)
C4—C11—C12—O1	−3.6 (10)	C23—C24—C25—C27	−178.9 (7)
C16—C11—C12—C13	3.2 (10)	C22—C21—C26—C25	−1.2 (10)
C4—C11—C12—C13	175.2 (6)	C5—C21—C26—C25	−178.2 (6)
O1—C12—C13—C14	175.6 (7)	C24—C25—C26—C21	−0.8 (10)
C11—C12—C13—C14	−3.2 (11)	C27—C25—C26—C21	179.7 (6)
C12—C13—C14—C15	−0.7 (11)	C24—C25—C27—C30	48.9 (10)
C13—C14—C15—C16	4.5 (10)	C26—C25—C27—C30	−131.5 (8)
C13—C14—C15—C17	−180.0 (7)	C24—C25—C27—C29	170.2 (7)
C12—C11—C16—C15	0.7 (10)	C26—C25—C27—C29	−10.3 (10)
C4—C11—C16—C15	−171.6 (6)	C24—C25—C27—C28	−70.4 (9)
C14—C15—C16—C11	−4.4 (10)	C26—C25—C27—C28	109.1 (9)
C17—C15—C16—C11	180.0 (6)		

### Hydrogen-bond geometry (Å, º)

**Table:**

*D*—H···*A*	*D*—H	H···*A*	*D*···*A*	*D*—H···*A*
O1—H1···N1	1.00 (10)	1.70 (10)	2.655 (7)	157 (8)
O2—H2···N2	0.95 (8)	1.83 (8)	2.656 (7)	144 (7)
